# Acceptability and fidelity of a psychosocial intervention (PROACTIVE) for older adults with depression in a basic health unit in São Paulo, Brazil: a qualitative study

**DOI:** 10.1186/s12889-021-12402-3

**Published:** 2021-12-14

**Authors:** Maiara Garcia Henrique, Maria Clara P. de Paula Couto, Ricardo Araya, Ana Vilela Mendes, Carina Akemi Nakamura, William Hollingworth, Pepijn van de Ven, Tim J. Peters, Marcia Scazufca

**Affiliations:** 1 LIM-23, Instituto de Psiquiatria, Hospital das Clínicas (HCFMUSP), Faculdade de Medicina, Universidade de São Paulo, São Paulo, Brazil; 2 Centre of Global Mental Health, Institute of Psychiatry, Psychology, and Neurosciences, King’s College, London, UK; 3 Department of Population Health Sciences, Bristol Medical School, University of Bristol, Bristol, UK; 4 Health Research Institute, University of Limerick, Limerick, IE Ireland; 5 Institute of Psychology, Friedrich Schiller University Jena, Jena, Germany

**Keywords:** Collaborative care, Task-shifting, Intervention, Depression, Older adults, Acceptability

## Abstract

**Background:**

Depression is a common condition in older adults, being often detected and treated initially in primary care. Collaborative care models including, for example, task-shifting and stepped-care approaches have been investigated to overcome the current scarcity of strategies and trained mental health professionals to treat depression. The PROACTIVE study developed a psychosocial intervention, which makes extensive use of technology in an intervention delivered mainly by non-specialists to treat older adults with depression. The aim of this qualitative study is to assess: 1. Health workers’ fidelity to the intervention protocol; 2. Acceptability of the psychosocial intervention from the viewpoint of older adult participants; and 3. Perceptions of the psychosocial intervention by the health workers.

**Methods:**

Qualitative methods were used to achieve our aims. The sample included participants (*N* = 31) receiving the intervention in the pilot trial and health workers (*N* = 11) working in a Basic Health Unit in the northern area of São Paulo, Brazil. Focus group, non-participant observation and structured interviews were used. Data were analysed using a thematic analysis approach.

**Results:**

1. Health workers’ fidelity to the intervention protocol: training, supervision and the structured intervention were crucial and guaranteed health workers’ fidelity to the protocol. 2. Acceptability of the psychosocial intervention from the viewpoint of older adult participants: Collaborative care, task-shifting, and stepped-care approaches were well accepted. The structured protocol of the intervention including different activities and videos was important to adherence of older adult participants 3. Perceptions of the psychosocial intervention by the health workers: It was feasible to have the home psychosocial sessions conducted by health workers, who are non-mental health specialists and received 3-day training. Training and supervision were perceived as crucial to support health workers before and during the intervention. Technology served as a tool to structure the sessions, obtain and store patient data, present multi-media content, guarantee fidelity to the protocol and facilitate communication among members of the team. However, extra burden was mentioned by the health workers indicating the need of adjustments in their daily duties.

**Conclusions:**

The PROACTIVE intervention was demonstrated to be feasible and accepted by both health workers and older adult participants. The qualitative assessments suggested improvements in training and supervision to ensure fidelity to protocol. To assess effectiveness a randomised controlled trial of the intervention will be conducted with the addition of improvements suggested by this qualitative study.

**Trial registration:**

The pilot study of which the present study gives support to was registered at the Brazilian Clinical Trials, UTN code: U1111-1218-6717 on 26/09/2018.

**Supplementary Information:**

The online version contains supplementary material available at 10.1186/s12889-021-12402-3.

## Background

Depression affects at least 10% of the older population and is a major challenge to health systems worldwide [1, 2]. The lack of timely recognition and treatment of late life depression undermines quality of life, and such impairments are comparable to those associated with serious physical illnesses [3]. To address these issues, the World Health Organization (WHO) recommends initiating treatment in primary care [1, 4, 5]. However, in some countries primary care is not fully prepared or resourced to embrace this task effectively. This is due to a lack of professionals, mental health stigma, and to the fact that depression is often not recognized, especially in older adults, being mistaken for signs of aging [1,5].

One model developed to overcome the treatment gap for late life depression in primary care is collaborative care [6–10]. This model consists of the cooperation of all members of the primary care team with support of trained mental health specialists to achieve the best outcome for patients [11, 12]. Collaborative care models can incorporate effective approaches such as: 1. Task-shifting (i.e., the transfer of duties from highly trained professionals to non-specialists or lay providers [13]); 2. Stepped-care (i.e., identification of those with greater need of a referral to more intensive treatments [14, 15]); and 3. Support of technology.

To date, collaborative care model interventions have been tested in different countries with positive results. However, in spite of that, previous studies have pointed out some challenges regarding its implementation. For instance, healthcare systems vary socio-culturally speaking, and contextual factors may influence successful implementation. Task-shifting, for example, pose as a good strategy in mental health interventions [16–18], but its acceptance by all professionals of the primary care team, and particularly by the non-specialist professionals, has been reported as a major challenge [14, 18, 19]. Health professionals claim it to be an extra work or perceive themselves (or are perceived by other team members) as not competent for the task [20]. A study carried out in the Netherlands used stepped-care to treat depression and the qualitative findings showed that it was accepted by participants but could be challenging when patients’ needs were not well identified, or when professionals were not prepared to deliver the best care and medical referral [14]. Although technology was of great use when delivering an intervention, professionals’ ability to use a device and application was a main concern, particularly in low-income countries [21].

Collaborative care, task-shifting, and stepped-care proved to be effective to help participants improve from depressive symptoms in high income countries, but few studies have tested these models in low- and middle-income countries [16, 17, 20, 22]. In Brazil, a recent study tested task-shifting to treat depression in primary care [22]; however interventions using collaborative care, task-shifting and stepped-care alongside technology to treat older adults with depression in primary care in Brazil still need to be tested. To meet this need, a new psychosocial intervention (PROACTIVE) was conducted with older adults with depression in the primary care in Brazil [23, 24]. The intervention consisted in health workers (nurse assistants and community health workers) delivering a psychosocial intervention with the help of an application on a tablet. This new intervention tested collaborative care and approaches such as task-shifting, stepped-care and the use of technology and showed to be effective in the reduction of depressive symptoms. Qualitative research methods play an important role during the development of this kind of programme to assess the acceptability and feasibility of incorporating these models into practice and suggest modifications, if needed [15, 25, 26]. Thus, using a qualitative approach, we conducted a process evaluation to investigate: 1. Health workers’ fidelity to the intervention protocol; 2. Acceptability of the psychosocial intervention from the viewpoint of older adult participants; and 3. Perceptions of the psychosocial intervention by the health workers.

## Methods

This study used qualitative methods to evaluate a new psychosocial pilot intervention targeting depression among older adults in socioeconomically deprived areas of São Paulo, Brazil [23]. The pilot study adhered to CONSORT 2010 guidelines and was registered at the Brazilian Clinical Trials, UTN code: U1111-1218-6717.

### The psychosocial intervention: the PROACTIVE programme

PROACTIVE is a standardised collaborative care psychosocial intervention [24]. It was developed to meet the needs of older adults registered with the Family Health Strategy (FHS), which is a primary care model within the Brazilian Unified Health Care System (SUS in Portuguese). The FHS covers approximately 60% of the population, mostly in socially deprived areas. The FHS is delivered through Family Health Teams (FHTs) that have a similar composition all over the country (one family doctor, one nurse, two nurse assistants and 6 community health workers). Each FHT is responsible for a catchment area of about 4000 individuals [27]. The teams work collaboratively, all members of the team meet weekly to discuss cases and plan treatment for patients.

PROACTIVE’s development was based on well-known models and approaches such as collaborative care [10, 11] (where an inter-professional team manages cases together and follow a structured plan of sessions to follow-up each participant), stepped-care [15], and task shifting [13]; in addition, the use of technology was incorporated. Non-specialist health workers such as Nurse Assistants (NAs) and Community Health Workers (CHWs) employed by a Basic Health Unit (UBS in Portuguese) were selected to deliver a 17-week home-based psychosocial intervention. Whilst other members of the primary care team collaborated with support tasks (The work was carried out in close collaboration with other members of the FHT. In weekly meetings, the team discussed the cases, the need for consultation with the family doctor and referrals as well as medication prescription), whenever needed. The PROACTIVE home sessions last about 50 min, in which the health worker uses a software application (app), developed by the researchers, installed on a tablet computer [28] to access a structured intervention protocol, composed by a pre-specified health assessment, and a list of activities and educational videos about depression and behavioural activation [29]. The data collected during the home session is stored in the tablet and can be used to discuss cases with other team members in the weekly meetings. All the information captured by the app is stored in a server accessible to the programme manager and clinical supervisors. An automated notification system is used to notify the manager of the UBS about older adult participants’ improvements or need for extra care based on the depressive symptoms assessed every session. Participants receive a booklet where they can read about depression and other contents of the intervention, write down the activities planned to be done between sessions, and write reminders of date and time of the next sessions.

The intervention is structured in two phases. During the initial phase, all older adult participants receive three weekly sessions; in the second phase, they are assigned, automatically by the app, to either low or high intensity treatment depending on the level of depressive symptoms at the 2nd and 3rd sessions, assessed with the Patient Health Questionnaire-9 (PHQ-9) [30]. The low intensity regimen has five additional sessions, being three sessions every other week and the last two, monthly. The high intensity regimen has eight additional sessions, being six sessions weekly and the last two, monthly. All sessions follow the same structure: 1. Start of session (rapport and checking “home activity”); 2. Collecting information (depression assessment, health questionnaire and mood assessment); 3. Giving information (videos: psychoeducation about depression, health and preventing accidents, behavioural activation); 4. Activities during the sessions (vicious and virtuous cycle of depression, list of pleasant activities); 5. Activities between sessions (“home activity”, planning activities for the week); and 6. Closure (summary of session) and scheduling next visit.

NAs and CHWs received a 3-day training course (total training time, 24 h) before the start of the psychosocial intervention. They also received weekly group supervision at the UBS, conducted by a psychologist, to discuss cases, difficulties following the protocol of the intervention and the use of the app, which also served as an ongoing training. In between supervisions, they had a support group on “WhatsApp” to reach the clinical supervisor (psychologist) in case of doubts or need of technological support. The rest of the team (nurses and doctors) received a mental health training given by a psychiatrist, who was remotely available in case the doctors needed any further support.

The following topics were addressed during the 3-day training course (lecture and role play):

1. Health worker interaction with the older adult participant during the home session: empathy towards the older adult participants’ needs, ability to conduct the intervention (for example, talking naturally versus reading the script on the app; understandable explanation of the intervention), openness to discuss emotional issues raised by the older adult participants during the session.
2. Addressing psychoeducation about depression and behavioural activation: helping patients to identify and engage in pleasant and meaningful activities, encouraging them and planning together.
3. Health worker ability to use the app (and its functions) installed on the tablet and the booklet: review previous sessions, assess depression and health conditions, play videos, activity planning and scheduling the sessions on the tablet.
4. Management of the time during the session: manage time to deliver the whole session, follow the structure of the sessions, and focus on the main topics and contents of each session.

#### Study setting

The present study was carried out in the single UBS allocated to the intervention arm of the pilot trial, located in the Northern area of the São Paulo municipality, Brazil. This UBS is affiliated to the FHS of the SUS and has seven FHTs.

#### Participants

The participants were health workers and older adults from the intervention arm of the pilot study.

- Health workers: Three nurse assistants (NA) (one from each FHT) and eight Community health workers (CHWs) (2 from each remaining FHTs) delivered the psychosocial intervention. All health workers were women. Although NAs and CHWs perform different activities in the UBS, the first assist the nurses with health procedures and vaccination and the latter visit the households to collect health information about the family members and communicate it to the FHT, they performed the same role in the psychosocial intervention. NAs have at least secondary education and an additional technical course on assisting nursing. CHWs have at least secondary education but no formal education (or training) on health and are residents in the UBS’s catchment area. Before starting the health workers’ training, the coordinator of the study explained the steps of the psychosocial intervention to the UBS manager, and the tasks health workers were expected to perform during the intervention. Like other UBS programmes, it was up to the manager to select which specific health workers among the CHWs and NAs would be a good fit to this type of intervention considering the specific tasks they would need to carry out. However, the coordinator of the study guided the manager to discuss also with the FHTs which CHWs and NAs would be available and/or willing to participate.
- Older adult participants: The sample comprised older adults included in the intervention arm of the pilot study. To be eligible for participation, they should be 60 years and over (in Brazil people 60 years old and over are considered elderly according to the Brazilian Statute of the Elderly [31]) and registered with the UBS while not presenting any exclusion criteria (complete deafness; terminal illness; psychotic symptoms; risk of suicide; inability to communicate due to cognitive impairment or other health problem). Older adults who were eligible were screened for depression symptoms (PHQ-9 > 9) and included in the sample in case of a positive screening. The final sample comprised 31 older adults (8 males, 23 females; see Table 2 for details).

#### Procedures

Each of the three study aims were tailored to allow for assessment of specific components of the intervention. A multi-method approach was therefore chosen to enhance the rigor of the study (see Table 1 for a description of the procedures for data collection and analyses for each study aim).

**Table 1:** Methodology of each aim of the study

Aims	Method of data Collected	Data Analysis	Participants	Data collection method
Health workers’ fidelity to the intervention protocol	Field observation notes, checklist form, audio recording of the sessions	Comparisons of field notes with checklist, report	Health workers and older adult participants	Non-participant observation
Acceptability of the psychosocial intervention from the viewpoint of older adult participants	Structured questionnaire	Thematic analysis (Deductive thematic analysis)	Older adult participants	Individual Interview
Perceptions of the psychosocial intervention by the health workers	Notes, recording, and transcription	Thematic analysis (Deductive thematic analysis)	Health workers	Focus Group

##### Health workers’ fidelity to the intervention protocol

A trained qualitative researcher (MGH, psychologist, female) carried out the non-participant observations, a method in which the *researcher* does not participate actively, but just observes a situation without interfering [32]. The observation occurs in natural settings and allows the observer to assess non-verbal cues as well, thus having greater ecological validity. We observed 14 psychosocial sessions (14 health worker/older adult dyads), following the flow of the intervention: 3 sessions from the initial phase of the intervention, 5 sessions from the second phase/low intensity regime, and 6 sessions from the second phase/high intensity regime. Session numbers four and six of the high and low intensity regimens had the same contents, for this reason we only observed sessions four and six of the low intensity regime. All health workers were observed at least once, and three of them were observed twice to cover all 14 sessions. Before asking consent to conduct the observation, the qualitative researcher (MGH) explained she was going to observe the entire session, take notes and audio record the conversation with a smartphone. The researcher and the health workers were already acquainted since the training. The session and the observation started after consent was granted for audio recording from both health worker and older adult participant. Data were collected during the sessions, using a checklist describing the structure of each session (Additional file 1), field notes, and the recording of the sessions. To assess the fidelity of health workers to the psychosocial intervention protocol, we analysed if the structure of each session was followed and if the health workers’ performance while delivering the home sessions was consistent with the four main topics covered during their training (interaction with the older adult during the session, addressing psychoeducation about depression and behaviour activation, ability to use the PROACTIVE app and booklet, and time management during the session).

##### Acceptability of the psychosocial intervention from the viewpoint of older adult participants

Individual interviews were conducted at the older adult participants’ home after completion of the intervention programme, around 26 weeks after the inclusion in the pilot trial. Three female qualitative researchers (MGH - psychologist, MS - PhD, MCPPC - PhD) conducted the individual interviews, at the final section of the pilot trial follow-up assessment. The interview consisted of a 22-question structured questionnaire (see full questionnaire in Additional file 2) and evaluates older adult participants’ views about the principles of the intervention (collaborative care, task shifting, stepped-care), and the structure of the psychosocial intervention (use of a standardized protocol, complex intervention delivered with the support of technology) and lasted about 30 min. Older adults’ answers to the questions were annotated, no recordings were done. Data were analysed by three members of the research team independently, grouping the answers into themes (deductive thematic analysis, that is, coding was based on the themes previously investigated – i.e., the guiding principles and protocol of the intervention) [33].

##### Perceptions of the psychosocial intervention by the health workers

All health workers who led the psychosocial intervention (3 NAs and 8 CHWs) participated in the focus group at the UBS at the end of the intervention. The aims of the focus group were explained, and consent was sought for audio recording. The focus group lasted approximately two hours. Three qualitative researchers (MGH, MS, MCPPC) conducted the focus group, one observed and took notes of the discussion and two conducted the conversation guided by a list of topics prepared prior to the group (expectations, demands, support, mental health, intervention protocol, collaborative care, training/supervision and use of technology, Additional file 3). The focus group’s audio recording was transcribed verbatim. Then, the three qualitative researchers team read the transcripts separately and analysed the data. Answers to the topics investigated were organised in thematic categories in a spreadsheet (Excel), and the data was analysed using a thematic analysis approach, where similar themes are separated into code frames [34]. The coding was based on a priori investigated themes (i.e., deductive thematic analysis) and some emerging themes from the data. Inter-rater reliability was achieved by having three researchers reading and coding data till disagreements were solved and no new themes were found.

## Results

### Health workers’ fidelity to the intervention protocol

The structure of the sessions was followed by all health workers according to the checklist. Below we describe the performance of the health workers based on the four main topics addressed during their 3-day training:

#### Health workers’ interaction with the older adult during the home sessions

In general, health workers interacted well with the older adult participants. Both CHWs and NAs showed empathy towards their problems and tried to meet their needs during the sessions. The first four sessions observed (session 1, 2, 3 of the initial phase, and session 4 of the second phase) were the most challenging regarding the interaction between health workers and older adults. During the first minutes of these sessions, some health workers (*n* = 4) were not completely at ease, i.e., they read rigorously the scripts on the app instead of being spontaneous in the conversations with the older adult participants. This was more evident with health workers observed conducting the first and second sessions of the initial phase of the psychosocial intervention. In some occasions, other members of the family were around while the sessions were being delivered and observed. But third parties did not interfere in the session.

During the first session observed, the health worker appeared anxious, sometimes talking too quickly or without eye contact with the older adult. The health workers had difficulty understanding when the participant indicated with gestures that they had not fully understood the content of the first video. As the home sessions progressed, we observed the health workers were more relaxed (especially after the initial phase, i.e., after the third or fourth session) and were able to have a more natural conversation with the older adults.

##### Addressing psychoeducation about depression and behavioural activation

Psychoeducation about depression was presented to older adult participants with the assistance of the animated videos and the booklet. No problems were observed, health workers played the videos and, whenever needed, gave more explanations with the help of the scripts on the tablet. The steps of the behavioural activation technique were introduced to older adult participants at different stages of the intervention. During the first three sessions (first phase), the health workers explained and showed to older adult participants, on the PROACTIVE app, the virtuous cycle of depression (e.g. If you do..., your low mood is likely to improve) and the vicious cycle of depression (e.g. If you do not do anything, your low mood is likely to get worse). In these sessions, the older adult participant was invited to play with the app, filling in the gaps in the cycles of depression to understand that what they do (or not) can help improve (or not) their mood. Some health workers (*n* = 3) only gave the oral explanation to older adult participants without showing the cycles on the app, as they had been trained. However, we observed that the verbal explanation was adequate and sufficient since participants demonstrated their understanding about the basic principle of behavioural activation. From the 4th session (second phase), the health workers motivated older adult participants to increase the level of pleasant and meaningful activities they do during the week. Health workers were trained to show to older adult participants a comprehensive list of activities in the app and ask them to choose the activities they wanted to do between sessions, without interfering with their choices, but helping them to plan how to achieve realistic goals. Only on one occasion (4th session observed), the health worker suggested the activity instead of asking what the older adult participant wanted to do. Health workers were also trained to discuss with older adult participants, at the beginning of all sessions, if the planned activities were performed and if there were any problems in carrying them out.

#### Health worker ability to use the app installed on the tablet and the PROACTIVE booklet

All health workers (*n* = 11) were able to log in to the app and use the app functions. In all sessions, older adult participants were assessed for depressive symptoms. Health workers were trained to ask the questions aloud to older adult participants or let them use the app to answer the questions. Both strategies worked well, and the information about the level of older adult participants’ depressive symptoms was collected in all sessions observed. None of the health workers skipped the videos or experienced problems to play them. At the end of all sessions, health workers reviewed the session with older adult participants. One of the functions of the app is to produce a script with a summary about each session. We observed that health workers read these scripts before reviewing the sessions with older adult participants. The health workers did not show any difficulties to use functionalities of the app to schedule the next session (date, time place), finalise the session, and synchronize the app with the study server. At the end of the sessions, health workers needed to write the date of the next session and the activities planned to be carried out between sessions in the PROACTIVE booklet. In four sessions observed, the health workers (*n* = 4) forgot to write down these notes in the booklet of the participant.

#### Management of the time during the session

Most sessions observed were completed within one hour. Health workers were able to go through all parts of the sessions, perform the activities with older adult participants, and discuss emotional and other issues raised by the older adult participants, without extending the length of the session. Only on one occasion (session 3 – first phase) was the health worker unable to complete the session in one hour. In that session, the health worker had difficulty keeping the focus of the conversation on issues related to the session.

### Acceptability of the psychosocial intervention from the viewpoint of older adult participants

Thirty-one older adult participants were interviewed using a structured questionnaire. Three quarters of those interviewed were female with half of the participants between the ages of 70 and 79. The vast majority had less than 8 years education and 75% had no income or received the equivalent of one Brazilian minimum wage (Table 2).

**Table 2:** Characteristics of older adult participants

Older adult participant identification number	Age	Gender	Education (years)	Income (BMW*)	PHQ-9 score at baseline	PHQ-9 score at follow up
P1	64	Male	0	1	13	4
P2	64	Female	4	1	13	1
P3	65	Female	0	no income	11	15
P4	71	Male	4	2-3	14	2
P5	70	Female	6	1	10	10
P6	61	Female	4	1	15	6
P7	65	Female	4	no income	16	0
P8	64	Female	12	1	12	0
P9	66	Male	7	1	19	8
P10	78	Female	4	1	16	10
P11	70	Male	11	1	13	3
P12	69	Female	5	1	13	3
P13	63	Female	5	1-2	15	0
P14	81	Female	0	1	15	0
P15	75	Female	2	1	12	5
P16	72	Male	0	1	16	1
P17	78	Female	4	1-2	18	8
P18	70	Female	4	1	16	0
P19	88	Male	0	1-2	12	0
P20	66	Female	11	1	13	6
P21	68	Female	8	2-3	17	1
P22	72	Female	4	1	25	2
P23	71	Female	7	1	18	10
P24	76	Female	2	1	19	6
P25	83	Female	4	1-2	20	6
P26	76	Male	4	1	13	0
P27	71	Female	4	1	16	4
P28	72	Male	0	1-2	13	3
P29	80	Female	0	1	12	4
P30	76	Female	0	1	13	1
P31	67	Female	0	1	19	9

*BMW = Brazilian minimum wage; 1 = BRL 788.00 ~ USD 242.46 American dollar at the time of the study (2016)

Below we present the older adult participants’ views of the psychosocial intervention grouped by the main themes explored in the interview. The a priori codes referred to the approaches implemented in the psychosocial intervention: Accepting the non-specialists, collaboration among the team, extra care and referrals, and technology as a tool to motivate and guide the sessions. Standardized protocol and other comments emerged as a posteriori codes from the analysis:

- Accepting the non-specialists: Older adult participants were aware that non-specialist health workers delivered the sessions, but that did not come as a barrier, on the contrary, they liked and felt comfortable talking to them. The majority of older adult participants (*n* = 29) had positive comments about the CHWs and the NAs work:

P12: *“I felt very comfortable with the CHW”;* P13: *“She [NA] is very kind and I feel comfortable talking to her”;* P14: *“When she [CHW] arrived I was filled with joy”;* P22: *“It is as if we [her and the CHW] were close friends”;* P30: *“She [CHW] is really nice, polite and explained everything to me”.*

Two older adult participants were not confident with the competence of the health workers to deal with severe cases of depression. Task-shifting was still accepted except for severe cases.

P6: “*Being acquainted to her [CHW] was really good and we had great conversations but there are things that only a psychologist knows”;* P5: “*In more severe cases, I guess it is important to have a specialized professional to guide the older adult participant, instead of a CHW”.*

- Collaboration among the team: Most older adult participants (*n* = 22) observed that the communication between the health workers and other team members resulted in improvements in the care they received from the FHT.

P18: *"They invited me to be part of the groups and do handcrafts there at the clinic. They are very affectionate with me.";* P8: *"Yes, my CHW talked to the team about my sleeping problems and now I am taking amitriptyline.";* P22: *" It changed a lot, they talked to the doctor about the appointments, and they treated me more carefully".*

A minority of older adult participants (*n* = 9) did not see any difference in the way they were treated at the clinic.

- Extra care and referrals: Collaboration between health workers and other team members was important in identifying older adult participants who needed to be seen by the family doctor. Six older adult participants were seen by the family doctor as result of this collaboration.

P8: *“I asked (to the CHW) for a consultation and I got it”;* P22: *“It [the sessions] helped me a lot, I was seen by the doctor and now I take sertraline*”; P23: *“The CHW asked for the doctor to see me, I got a consultation, and the doctor changed my medication, it helped me”.*

- Technology as a tool to motivate and guide the sessions: All older adult participants enjoyed the use of the tablet during the sessions:

P20: *"I thought it was good, it was motivating and explained a lot of things";* P14: “*It is better with the tablet, that way every information is there and can be showed to us*”; P18: *“The CHW was showing me the things on the tablet, it made it easier to understand and learn more”;* P10: *“I think the tablet helps a lot, it makes us focus on what is being discussed.";*

They also enjoyed watching the videos, which were motivating and educative:

P25: *“I liked the videos, they cheered us up and taught us many things”;* P13: *"The animation is an imitation of me and the health worker, she tells me what to do to feel better";* P21: *"You can understand it, it is simple, Sum, Sani and another colleague [Sum, Sani and the colleague (Nê) are characters from the video animations] want us to understand the situation and teach us solutions to the problems".*

- Standardized Protocol: The majority of older adult participants (*n* = 26) expressed that having a structured protocol and different activities in each session was important for adherence to treatment, others were neutral about it:

P9: *"I thought it was good, because if it was open [no structure], we would not know what to say";* P6: *"I thought it was good and even with the structure, I had space to talk and it was good to know that there is not only one thing, but several things to do [during the sessions]. It was interesting";* P20: *"I thought it was good and interesting. When you have multiple activities, it helped to keep me interested";* P23: *"It was never tiring, it was good to have so many different activities and learn something new each day”;* P13: *"I liked to answer questions, talk, and do activities on the tablet because I learned a lot".*

One older adult participant did not like the structured protocol, mainly because she had to answer many questions every session:

P17: *"It's kind of tiring. Every time the same thing.”.*

Five participants reported having difficulties to do the activities between sessions, but all remembered choosing and planning the activities.

*Other comments about the intervention*

- Twenty-five older adult participants found the number of sessions sufficient, five expressed a desire to continue the sessions, and one older adult participant felt the number of sessions was more than necessary. Of the 31 older adult participants interviewed, nine (29%) discontinued the psychosocial intervention before the end of the sessions (five during the initial phase and four during the second phase, two from each intensity, low and high). Some of the reasons for discontinuing the sessions were: misgiving CHWs’ skills, believing they did not need treatment, not having time to participate or commit to the activities.

P5: “*I was not really sure about the treatment with the CHW, she is not a psychologist*”; P9: *"I do not know why I told her not to come. I thought that I was bothering her and that I would not understand the questions. I regret it. Now I started doing the activities she was telling me";* P17: “*I did not need a treatment and I thought I was going to take someone else’s opportunity”;* P28: *“I used to go out a lot, the health worker couldn’t find me so I asked her to stop coming, but now I have time”.*

Eight participants reported never using the booklet.

### Perceptions of the psychosocial intervention by the health workers

All health workers that conducted the psychosocial sessions (8 CHWs and 3 NAs) participated in the focus group. They were women, six (55%) aged between 29 and 34 years old, three (27%) between 35 and 45 years old, and two (18%) between 50 and 60 years old. The results presented below are organised in four thematic categories, reflecting the main themes defined a priori: (1) Capability of non-specialists, (2) Training and clinical supervision, (3) Team collaboration, and (4) Impact of the intervention in their work routine at the UBS. Within each of the thematic categories, the following a posteriori codes emerged from the narratives: Feeling capable of delivering the intervention (Capability of non-specialists), feeling supported and prepared (Training and clinical supervision), involvement of other members of the Family Health Team in handling cases (Team collaboration), and professional and institutional impact (Impact of the intervention in their work routine at the UBS).

#### Capability of non-specialists

- Feeling capable of delivering the intervention:

In general, the health workers did not experience problems delivering the programme and felt welcomed by the older adult participants. Only one CHW (CHW6) mentioned that the older adult participants she visited, refused to participate in the psychosocial sessions because the CHW was not a mental health specialist.

CHW 1: “*Of course! I am sure [that a CHW could deliver the intervention]”;* CHW 3: *“We were well trained; we can do this [intervention]”.*

CHW 6: “*There was only one older adult participant that I was seeing who was hesitant and gave up.”.*

#### Training and clinical supervision

- Feeling supported and prepared:

Health workers perceived the training received and the group supervision as opportunities to talk about difficulties and support their work. In addition, supervision was also perceived as a space to share experiences among health workers, learn from each other, and discuss difficulties related to the use of the PROACTIVE app during the home sessions.

CHW 8: *“So, it was a support [for us], because we were afraid of what could happen [in the sessions] … What now? Then, we had the supervision to talk; they [supervisors] were there to help with our questions”.*

NA 2: *“And then, in the supervision, everyone’s experiences counted, what they were going through [with the older adult participants] … Hence, I think we could get a little bit of all the experiences, you know?”.*

#### Team collaboration

- Involvement of other members of the Family Health Team in handling cases:

Health workers perceived their participation in the PROACTIVE programme as a facilitator of the communication with FHT members. One of the results of the collaboration between health workers and FHT’s doctors and nurses was the involvement of these health workers with some older adult participants who were receiving the psychosocial intervention:

CHW 4: “*I talked to the CHW – ‘Did you notice this and that?’ and she goes like – ‘No, but one day I noticed this other thing and you?’, then we started putting pieces together until we decided to gather with the FHT to have a different look at this older adult participant; there was something different, you know?”*

CHW 3: *“Yes, the doctors, nurses [were collaborative], they got involved, they referred older adult participants, they followed [the older adult participants] closely; in team A, the doctor went to the older adult participant’s house [for a consultation] and after that the older adult participant came to the UBS for health exams”.*

#### Impact of the intervention in their work routine at the UBS

- Professional impact:

All health workers indicated that participation in the PROACTIVE programme had a positive impact on their work at the UBS, as they acquired new skills.

CHW 8: *“Today, even without the tablet and not being part of the intervention programme any longer, we can still offer some counselling to people.”;*

CHW 6- *“Everything that we learned, that opened our mind, that helped in our work was valid. Every day we learn something, we change our point of view… This [the intervention programme] came to intensify it, to change our view”.*

- Institutional impact:

The extra work associated with participating in the PROACTIVE programme was a hot topic during the focus groups. The main complaint was related to the difficulty of managing the time to perform the routine tasks at the UBS with psychosocial sessions at home, all within working hours. The home sessions were added to their other tasks at the UBS. They also felt the time spent with the PROACTIVE programme (sessions and supervision) was more than what they expected initially. Health workers also complained about frictions with colleagues over the time they spent on the PROACTIVE programme.

CHW 1: *“Yes, because it was an extra burden, wasn’t it? Our usual tasks never stopped, but it [the programme] was something, it left a good taste in my mouth…”.*

NA 3: *“Much more, much more time than we have imagined... But it was good”;* CHW 7: *“I didn’t know it was going to be that much”.*

CHW 4: *“There were frictions, you know? Because we did not do other things, we ended up taking that time to do these other things we had not planned… So, there were divergences”.*

## Discussion

This study assessed the acceptability and fidelity of the PROACTIVE psychosocial intervention for treating older adults with depression in basic health units in Brazil using qualitative research methods. The main findings indicated that (1) task-shifting in this context was acceptable (i.e., older adult participants accepted having the intervention delivered by non-mental health specialists, and health workers were able of delivering it as defined in the protocol), (2) collaborative care and stepped-care ensured adequate treatment, and (3) technology was crucial for fidelity to the protocol and motivating participants.

Overall, the training and supervision provided to the health workers before and after the psychosocial intervention achieved its goals [35]. The intervention protocol was applied properly by the health workers with minor possible improvements to be addressed, and they did not have difficulties using the PROACTIVE app during the home sessions. Although most health workers complained about the extra work associated with their participation in the programme, they valued the experience and felt the programme was needed for treating older adults with depression.

Since collaborative care is already part of the FHTs work, it was not something completely new to health professionals at primary care. However, with PROACTIVE, non-specialized health workers had more space to talk at meetings and could contribute to the teams’ discussions when dealing with depression cases, which was seen as empowering for them. Thus, the programme adapts well to the existing FHS, which functions similarly across Brazil in that staff cooperate with each other when dealing with patients in their coverage area. Hence, the pilot study suggests that the PROACTIVE programme is likely to be acceptable and feasible all around the country. Sometimes, patients may need more or less care depending on symptoms or other reasons [14], for that reason stepped-care was another component in this intervention. Accordingly, patients who did not improve were referred to the family doctor and when needed, to a specialist, but they were only identified with the help of continuous depression assessments during weekly sessions and the automated notifications to the FHT.

As regards task-shifting, prior to the study three main concerns were identified: 1. whether non-mental health specialists would be able to deliver the intervention; 2. whether the intervention would burden care workers with extra tasks; and 3. whether older adult participants would accept this kind of intervention and its delivery by non-mental health specialists. Some studies in Africa using lay providers in primary care have shown that the lack of competence in specific tasks was a great challenge resulting in risks to patients [16, 17]; however, these studies included procedures that needed more specific training and supervision and were not related to counselling or mental health. Another study, also in Africa, used task-shifting in mental health and proved to be effective when there is enough training and support throughout the intervention [20]. PROACTIVE showed that non-mental health specialists (health workers) felt capable of delivering the intervention with the assistance of the information contained in the tablets, and enough training and supervision to be compliant to the protocol. It is interesting that the health workers observed in the last sessions were more secure of themselves when delivering the protocol and were able to have a natural conversation instead of simply reading the script on the tablet.

A challenge known in task-shifting is that lay providers may be overwhelmed with competing tasks [17, 20], which is consistent with our findings. Health workers assumed a new role, but their other duties were not adjusted, and they did not feel supported by their teams. Although, this is an issue in a study context, outside that context they would be allowed to manage their own schedules, choosing the quantity of patients they can see at a time to avoid overlapping tasks.

In spite of older adult participants’ adherence to the intervention and its materials, their high attendance and satisfaction with the intervention, the dropout rate was around 29%. Some of the reported reasons for dropping out were not thinking treatment was needed (lack of information about depression and difficulty in perceiving themselves depressed), lack of time, and doubting the competence of the CHW to deliver a psychosocial intervention targeting depression. It will be important to develop strategies, such as more training and psychoeducation to reduce mental health stigma and increase awareness of the importance of treatment in order to reduce the dropout rate. Notwithstanding for the participants who completed the psychosocial intervention the fact that it was delivered by a non-mental health specialist was not an issue in terms of trust, bonding, or acceptability of the intervention, some participants did indicate that this represented a barrier for their adherence. This finding is in contrast with previous studies, which showed that having non-specialists delivering an intervention is usually well accepted and even preferred by patients. In spite of that, this finding should be taken into consideration for the development of the future trial in terms of discussing strategies that can decrease the resistance of having non-specialists in this role [36]. Issues related to privacy may have played a role in not accepting the non-specialists’ care. CHWs live in the same neighbourhood and NAs are health workers with whom the older adult participants have close contact in the UBS. At the same time, that close contact was also referred by other participants as a positive point when bonding with CHW during the intervention, they feel more comfortable with them. Another crucial point was face-to-face sessions possibly impacting positively on participants’ adherence to the PROACTIVE psychosocial intervention. CASPER study, another collaborative care intervention in the United Kingdom [18], in which psychologists and nurses delivered sessions by telephone showed that although results in the improvement of depressive symptoms were positive, patients reported preferring face-to-face sessions instead of telephone sessions [18, 19].

The use of technology is known to be challenging in LMIC [21] due to professionals or patients’ lack of ability to work with technological devices. In the PROACTIVE programme the older adults do not use tablet computers themselves, but with the support of the health workers, who use the device during sessions. Therefore, from the perspective of the older adults the use of technology was not an issue. In fact, they benefited from the technology, as a large proportion of the older adult participants are illiterate, and the use of videos stored in the tablet allows easy access to information. Another important issue is mental health stigma and patients’ difficulty in accepting the depression diagnosis, therefore not seeking professional help [37] or withdrawing from the intervention because they did not understand the need for taking part in it [18, 38]. In the PROACTIVE programme the video animations helped with these problems as older adult participants reflected on the characters, learnt from them, and felt motivated in adhering to the treatment. From the health workers’ perspective, technology was of great use to structure the intervention, record and store older adult participant responses and present intervention content, and they did not struggle using it. The results of our study indicate that the training was sufficient to teach them how to manage the tablet computer and work with the app. The scripts on the tablet were of great use for health workers, allowing an adequate session delivery and fidelity to the protocol.

Supervision allowed us to provide ongoing training to the health workers and implement improvements during the pilot intervention. The functionality of the app on the tablet, for instance, was frequently adapted to resolve difficulties the health workers faced, such as problems accessing data on the tablet or accidentally skipping important content. The qualitative findings contributed to suggest improvements for the training and supervision in the main RCT, such as highlighting the importance of older adults to engage in pleasant activities (behavioural intervention) and strengthen their autonomy when dealing with depressive symptoms and choosing their own activities. Also, some minor issues in fidelity must be addressed, such as not entering data in the app, skipping videos or activities and managing time poorly. A way to avoid skipping content or lacking entrance of data is to block the screen in the app and add a reminder. Time management also needs more attention and must be discussed in the training and role played with health workers, raising strategies to keep the focus in the intervention. These issues should also be raised in weekly supervisions to ensure fidelity. Another improvement suggestion would be to have more training meetings with other members of the team (doctors and nurses) from time to time to address the overlap of tasks for non-specialists. As expected, it was confirmed that the psychoeducation texts in the booklet were not useful for illiterate older adult participants, and sometimes health workers forgot to use them, so the booklet will be reconsidered or highlighted in training as a useful tool to plan activities.

### Limitations

Although this qualitative study enhances our understanding of the acceptability and fidelity of the PROACTIVE psychosocial intervention, it does so with some limitations that should be considered when interpreting results.

First, non-participant observations were carried out only once for each session, moreover health worker and the older adult participant (dyad) were observed once in a specific session, not allowing us to assess improvements with gained experience along the intervention. Future qualitative studies should observe the same health worker conducting sessions at different time points to have a better overview of their development over time. We think that the focus group with the health workers conducted at the end of the intervention programme may have helped to reduce this issue though, as they have reported their experience conducting the intervention.

Second, even though during the non-participant observations the observer behaved unobtrusively, we have to concede that the presence of the qualitative researcher during the observations may have influenced the way the health workers behaved. Health workers reported feeling anxious while observed. The fact that based on the narratives of older adult participants the intervention developed well may indicate that the anxiety felt by health workers while delivering the intervention was restricted to the session in which they were observed. Observing sessions at different time points would maybe have mitigated the effect related to the observer’s presence in the session.

Third, the structured interviews could have been recorded, allowing checking back on data during the analysis process. However, participants’ concern for privacy was prioritized and the research team attempted to overcome this issue by registering carefully and in a standardized way all answers and relevant details. Additionally, there were quantitative questions in the structured interview that we have not analysed. We have used a structured questionnaire, which is an approach that has the advantage of being standardized since every participant answers the same questions. Nonetheless, we are aware that this type of approach has limitations for qualitative investigations, offering less opportunity for participants to fully express themselves and for the researchers to explore broad areas of interest in more depth.

Fourth, a de-identified survey could have been conducted in addition to the focus group to capture a greater range of perceptions from the health workers, since some of them may not have spoken-up in the focus group.

Last, the qualitative assessment was conducted in the pilot phase, which means that the sample size was defined by the pilot design, which for pragmatic reasons, included a maximum of three participants per health worker, and 11 health workers. Therefore, we did not use saturation as a criterion to stop data collection. Previous studies emphasized the importance of having a sample that allows identifying meaningful patterns across a dataset. Recommendations on sample size vary though with at least six participants being recommend for thematic analysis, for example. Hence, we believe our sample of 31 older adult participants with depression allowed us to generate meaningful qualitative insights regarding the pilot phase of the PROACTIVE psychosocial intervention [39].

Despite these limitations, our study provided useful insight on the acceptability and fidelity of the PROACTIVE psychosocial intervention. The qualitative findings attest to the good acceptability of the intervention by the older adult participants and to the fidelity to the intervention protocol by the health workers.

## Conclusion

Qualitative data obtained in the pilot study indicated that the PROACTIVE psychosocial intervention programme was acceptable to both older adult participants and health workers. Training and supervision were crucial to reach good fidelity to protocol and improve the ability of health workers when using technology. Technology itself also improved the fidelity to the protocol and reduced the need for longer training, as well as served as a tool to deliver information and notifications, allowing collaborative-care and stepped-care to work as intended. The structured protocol and behavioural activation achieved the expected aims, by teaching older adult participants about depression and giving them the autonomy to deal with symptoms, plan and undertake positive action to improve their mental healthiness, and prevent relapse. Further adjustments in the intervention will be made to adapt the protocol according to the findings in this study. This pilot study also presented quantitative results [23] showing a more significant reduction in depression symptoms (PHQ-9) in the intervention arm. Qualitative and quantitative results were analysed independently. However, they point in the same direction, namely: that the intervention is feasible; it is likely to reduce depressive symptoms and the guiding principles of the intervention are well accepted by the participants. To assess effectiveness, an RCT [40] is being conducted using the improvements suggested by the qualitative data in this study.

## Supplementary Information

**ESM 1.**

## Data Availability

The datasets generated during and analysed during the current study are not publicly available. This is a qualitative study and all the material used in the analysis are reports and not databases. However, the data are available from the corresponding author on reasonable request.

## Abbreviations

LMIC: low and middle-income countries
RCT: randomised controlled trials
UBS: Unidades Básicas de Saúde or Basic Health Units
FHS: Family Health Strategy
SUS in Portuguese: Brazilian Unified Health Care System
FHT: Family Health Team
NA: Nurse Assistant
CHW: Community Health Worker
App: Software application
PHQ-9: Patient Health Questionnaire-9
