# Supplementary material for: Acceptability and fidelity of a psychosocial intervention (PROACTIVE) for older adults with depression in a basic health unit in São Paulo, Brazil: a qualitative study
Source: BMC Public Health. 2021 Dec 14;21:2278. doi: 10.1186/s12889-021-12402-3 (PMC8670151; doi:10.1186/s12889-021-12402-3)
Supplement: Supplementary file 1 — ESM 1. [file 12889_2021_12402_MOESM1_ESM.docx]

**ADDITIONAL FILE 1**

**Checklist for non-participant observation of the sessions**

Instructions for the research assistant: For each session answer (YES/NO) if the health worker performed each activity planned and write any relevant observation about the quality of his/her performance. Answer the checklist while you observe the home session. Then, complete your answers/observations based on your annotations and listening the audio recorded during the session.

For all sessions, observe fidelity to the training given, based on the four topics below:

- - - 1. Interaction with the older adult during the session
      2. Addressing psychoeducation about depression and behaviour activation
      3. Ability to use the PROACTIVE app and booklet
      4. Time management during the session

| **INITIAL PHASE** | |
| --- | --- |
|  | **Session 1** |
|  | 1) start the session (engage with the patient) |
|  | 2) get information (PHQ-9, mood and health assessment) |
|  | 3) give information **(psychoeducation about depression and how to cope with it**) |
|  | 4) activities during the session (improving depression) |
|  | 5) home activity (planning activities to be done between sessions) |
|  | 6) end of session (review and schedule next session). |
|  |  |
|  | **Session 2** |
|  | 1) start the session (engage with the patient) |
|  | 2) get information (PHQ-9, mood and health assessment) |
|  | 3) give information **(psychoeducation about depression and how to cope with it**) |
|  | 4) activities during the session (simple strategies to cope with depression) |
|  | 5) home activity (planning activities to be done between sessions) |
|  | 6) end of session (review and schedule next session). |
|  |  |
|  | **Session 3** |
|  | 1) start the session (engage with the patient) |
|  | 2) get information (PHQ-9, mood and health assessment) |
|  | 3) give information **(psychoeducation about depression and how to cope with it**) |
|  | 4) activities during the session (simple strategies to cope with depression) |
|  | 5) home activity (planning activities to be done between sessions) |
|  | 6) end of session (review and schedule next session) and **explain second phase** |

| **SECOND PHASE** | | | | |
| --- | --- | --- | --- | --- |
|  | **Low intensity** (PHQ-9 < 10 in sessions 2 and 3) |  | | **High intensity** |
|  | **Session 4** |  | | **Session 4 (session not observed)** |
|  | 1) start the session (engage with the patient) |  | | 1) start the session (engage with the patient) |
|  | 2) get information (PHQ-9, mood and health assessment) |  | | 2) get information (PHQ-9, mood and health assessment) |
|  | 3) give information **(behavioural activation)** |  | | 3) give information **(behavioural activation)** |
|  | 4) activities during the session **(list of activities they like)** |  | | 4) activities during the session **(list of activities they like)** |
|  | 5) home activity **(Activities they like and already do, do them more)** |  | | 5) home activity **(Activities they like and already do, do them more)** |
|  | 6) end of session (review and schedule next session) |  | | 6) end of session (review and schedule next session) |
|  |  |  | |  |
|  | **Session 5** |  | | **Session 5** |
|  | 1) start the session (engage with the patient) |  | | 1) start the session (engage with the patient) |
|  | 2) get information (PHQ-9, mood and health assessment) |  | | 2) get information (PHQ-9, mood and health assessment) |
|  | 3) give information **(behavioural activation)** |  | | 3) give information **(behavioural activation)** |
|  | 4) activities during the session |  | | 4) activities during the session |
|  | 5) home activity **(Activities they like and already do, do them more)** |  | | 5) home activity **(Activities they like and already do, do them more)** |
|  | 6) end of session (review and schedule next session) |  | | 6) end of session (review and schedule next session) |
|  |  |  | |  |
|  | **Session 6** |  | | **Session 6 (session not observed)** |
|  | 1) start the session (engage with the patient) |  | | 1) start the session (engage with the patient) |
|  | 2) get information (PHQ-9, mood and health assessment) |  | | 2) get information (PHQ-9, mood and health assessment) |
|  | 3) give information **(behavioural activation)** |  | | 3) give information **(behavioural activation)** |
|  | 4) activities during the session |  | | 4) activities during the session |
|  | 5) home activity **(Activities they would like to do, start doing them)** |  | | 5) home activity **(Activities they would like to do, start doing them)** |
|  | 6) end of session (review and schedule next session) |  | | 6) end of session (review and schedule next session) |
|  | **Session 7** |  | | **Session 7** |
|  | 1) start the session (engage with the patient) |  | | 1) start the session (engage with the patient) |
|  | 2) get information (PHQ-9, mood and health assessment) |  | | 2) get information (PHQ-9, mood and health assessment) |
|  | 3) give information **(behavioural activation)** |  | | 3) give information **(behavioural activation)** |
|  | 4) activities during the session |  | | 4) activities during the session |
|  | 5) home activity **(behaviour they would like to change)** |  | | 5) home activity **(Activities they would like to do, start doing them)** |
|  | 6) end of session (review and schedule next session) |  | | 6) end of session (review and schedule next session) |
|  | **Session 8** | |  | **Session 8** |
|  | 1) start the session (engage with the patient) | |  | 1) start the session (engage with the patient) |
|  | 2) get information (PHQ-9, mood and health assessment) | |  | 2) get information (PHQ-9, mood and health assessment) |
|  | 3) give information **(behavioural activation)** | |  | 3) give information **(behavioural activation)** |
|  | 4) activities during the session | |  | 4) activities during the session |
|  | 5) **ndo fe of relapse prevention strategies** | |  | 5) home activity **(behaviour they would like to change)** |
|  | 6) end session and farewell | |  | 6) end of session (review and schedule next session) |

|  | **Session 9** |
| --- | --- |
|  | 1) start the session (engage with the patient) |
|  | 2) get information (PHQ-9, mood and health assessment) |
|  | 3) give information **(behavioural activation)** |
|  | 4) activities during the session |
|  | 5) home activity **(behaviour they would like to change)** |
|  | 6) end of session (review and schedule next session) |
|  |  |
|  | **Session 10** |
|  | 1) start the session (engage with the patient) |
|  | 2) get information (PHQ-9, mood and health assessment) |
|  | 3) give information **(behavioural activation)** |
|  | 4) activities during the session |
|  | 5) **present of relapse prevention strategies** |
|  | 6) end of session (review and schedule next session) |
|  |  |
|  | **Session 11** |
|  | 1) start the session (engage with the patient) |
|  | 2) get information (PHQ-9, mood and health assessment) |
|  | 3) give information **(behavioural activation)** |
|  | 4) activities during the session |
|  | 5) **present of relapse prevention strategies** |
|  | 6) end session and farewell |

**ADDITIONAL FILE 2**

**Structured questionnaire to assess patients’ understanding and perceptions of the protocol**

1 Do you know which professional (Nurse assistant, Community health worker or other) delivered the intervention to you?

2 How did you feel about being seen by that kind of professional?

3 Did you ever doubt this professional would be capable of delivering this kind of intervention?

( ) Yes ( ) No

**If yes:**

3.1 Did you change your mind after the sessions started?

( ) Yes ( ) No

1. During the intervention, have you had any medical consultation in the UBS?

( ) Yes ( ) No

**If Yes:**

4.1 Did you ask for a consultation or were you referred by the health worker?

4.2 Did the consultation helped you with the depression?

4.3 Were you treated any differently by the Health Team in the UBS after the start of the intervention? How was it?

**Over the last months you received some home sessions. In each session, the health worker talked to you about depression, simple strategies and showed you videos about those topics.**

1. The amount of sessions you received was:

( ) More than needed ( ) Less than needed ( ) Enough

1. What is your opinion about this kind of session, in which the health worker is guided by a *Tablet* with specific content for each session?

7. Did you feel you had time to talk about other subjects? What topics did you use to talk about the most?

1. Initially, the sessions were weekly, then they were every other week and at the end, monthly. Did those gaps in between sessions (intervals) have any impact in your treatment?

9. If you could choose, how would you like the time between session to be?

**Over your time in the PROACTIVE intervention, you have seen many things. Every session you were asked about your health and depression symptoms, then each session you watched different videos about depression, pleasant activities, behaviours to be changed; you also planned activities and at the end of the intervention you learned how to prevent relapse. You did all that using a tablet with the help of the health worker.**

1. What is your opinion about doing so many things in the tablet during the session?
2. Which sections or activities did you like to do the most? (talk about health, watch videos, activities in the session or home activity)
3. Do you remember talking about the ‘virtuous and vicious cycle of depression’?

( ) Yes ( ) No

**If Yes:**

What do you remember?

- 1. The ‘virtuous and vicious cycle of depression’ (it shows that when we are depressed and we don’t do anything about it, we get worse, but if we try a pleasant activity, we improve from the symptoms). Did you learn what to do to feel better?

( ) Yes ( ) No

1. Do you remember choosing activities to be done between sessions?

( ) Yes ( ) No

**If Yes:**

13.1 What is your opinion about doing activities between sessions?

13.2 Could you do the activities planned?

( ) Yes ( ) No ( ) Sometimes

13.3 Whenever you could not do (if so) the activities, what were the main reasons not to do them?

( ) Financial

( ) Mobility

( ) Family issues

( ) Health issues

( ) Other. Which?

1. Do you remember answering questions about your symptoms?

( ) Yes ( ) No

**If Yes:**

- 1. Did those questions let you more aware of how you were?
  2. How did you feel about answering those questions every session?

1. You watched many videos over the sessions. Is there any video that you remember?
   1. What do you think about the character in the video animations?
   2. Do you remember the anchor (the woman talking)? Could you understand her?
2. You received a DVD with all the videos of the sessions. Do you think having the videos will be useful for you in the future?

**You received a booklet in the first session. Can I see that booklet?**

1. Did you use the booklet? ( ) Yes ( ) No
   1. Do you know how the booklet works and what it is for?

**Check the booklet:**

- 1. Are the activities planning sheets filled?

( ) Yes, completely ( ) Yes, partially ( ) No

- 1. Is the scheduling written down?

( ) Yes, completely ( ) Yes, partially ( ) No

**To wrap up, I would like to ask you some things about the intervention.**

1. Do you think the home sessions were important to you? Why?
2. What is the main thing you learned with the session?

20 Comparing yourself before and after the sessions, how do you feel now? Are you better from depression?

**Only for those who did not receive any session:** Some months ago, you were interviewed by a researcher and invited to participate in the intervention. What made you change your mind when the health worker reached you to start the home sessions?

1. **Only for those who interrupted the intervention:** You have interrupted the home sessions, why is that?
2. When you decided not to participate anymore, did you receive a DVD with a letter? Who delivered to you?
3. How do you feel about having received the intervention in your house (or UBS if that is the case)?
4. Do you think you can use what you have learned in the future?

Suggestions, complaints, doubts or comments.

**ADDITIONAL FILE 3**

**Guiding topics of the focus group with health workers**

| **Topics raised during the focus group** |
| --- |
| - **(Expectations)** Expectations about the PROACTIVE before starting the home sessions. - **(Demand/burden of the programme)** If the time expend with the PROACTIVE was the expected. Problems dealing with other tasks at the UBS while delivering the intervention. Burden. - **(Institutional support)** Support received from other members of the UBS team and the manager during participation in the PROACTIVE. - **(Mental health care)** How they felt conducting home sessions to older adults with depression, motivations and challenges. If welcomed by patients. - **(Effectiveness of the intervention)** Impressions about the helpfulness/effectiveness of PROACTIVE for patients. Impact of the programme on patients’ views about depression. - **(Protocol)** If protocol was helpful to engage patient in the sessions/ activities. Views about the number of sessions and duration of the programmeme. - **(Collaborative-care)** Quality of the communication and collaboration with the UBS team during the intervention - **(Training and supervision)** Effectivity of the training, supervision and technical support (app and tablet) to conduct the intervention. - **(Use of technology)** Use of any form of technology (tablets, computers, apps) in other programme conducted at the UBS. Thoughts and difficulties. - **(Suggestions)** PROACTIVE’s impact on professional and personal life. Likes and dislikes. Suggestions for improvements. |

**ADDITIONAL FILE 4**

**COREQ (COnsolidated criteria for REporting Qualitative research) Checklist**

A checklist of items that should be included in reports of qualitative research. You must report the page number in your manuscript where you consider each of the items listed in this checklist. If you have not included this information, either revise your manuscript accordingly before submitting or note N/A.

| **Topic** | **Item No.** | **Guide Questions/Description** | **Reported on**  **Page No.** |
| --- | --- | --- | --- |
| **Domain 1: Research team**  **and reﬂexivity** | | | |
| *Personal characteristics* | | | |
| Interviewer/facilitator | 1 | Which author/s conducted the interview or focus group? | 10 |
| Credentials | 2 | What were the researcher’s credentials? E.g. PhD, MD | 8, 9 |
| Occupation | 3 | What was their occupation at the time of the study? | 8,9 |
| Gender | 4 | Was the researcher male or female? | 8,9 |
| Experience and training | 5 | What experience or training did the researcher have? | 8,9 |
| *Relationship with*  *participants* | | | |
| Relationship established | 6 | Was a relationship established prior to study commencement? | 8 |
| Participant knowledge of  the interviewer | 7 | What did the participants know about the researcher? e.g. personal  goals, reasons for doing the research |  |
|  |  |  | 8 |
|  |  |  |  |
| Interviewer characteristics | 8 | What characteristics were reported about the inter viewer/facilitator?  e.g. Bias, assumptions, reasons and interests in the research topic |  |
|  |  |  | n/a |
|  |  |  |  |
| **Domain 2: Study design** | | | |
| *Theoretical framework* | | | |
| Methodological orientation and Theory | 9 | What methodological orientation was stated to underpin the study? e.g. grounded theory, discourse analysis, ethnography, phenomenology,  content analysis |  |
|  |  |  | 9,10 |
|  |  |  |  |
| *Participant selection* | | | |
| Sampling | 10 | How were participants selected? e.g. purposive, convenience,  consecutive, snowball |  |
|  |  |  | 7 |
|  |  |  |  |
| Method of approach | 11 | How were participants approached? e.g. face-to-face, telephone, mail,  email |  |
|  |  |  | 9,10 |
|  |  |  |  |
| Sample size | 12 | How many participants were in the study? | 7 |
| Non-participation | 13 | How many people refused to participate or dropped out? Reasons? | 17 |
| *Setting* | | | |
| Setting of data collection | 14 | Where was the data collected? e.g. home, clinic, workplace | 9,10 |
| Presence of non-  participants | 15 | Was anyone else present besides the participants and researchers? |  |
|  |  |  | 11 |
|  |  |  |  |
| Description of sample | 16 | What are the important characteristics of the sample? e.g. demographic  data, date |  |
|  |  |  | 13, 17 |
|  |  |  |  |
| *Data collection* | | | |
| Interview guide | 17 | Were questions, prompts, guides provided by the authors? Was it pilot  tested? | 9, 10 Additional files 1, 2, 3 |
|  |  |  |  |
| Repeat interviews | 18 | Were repeat inter views carried out? If yes, how many? | n/a |
| Audio/visual recording | 19 | Did the research use audio or visual recording to collect the data? | 8, 10, 26 |
| Field notes | 20 | Were ﬁeld notes made during and/or after the inter view or focus group? | 9, 10 |
| Duration | 21 | What was the duration of the inter views or focus group? | 9, 10 |
| Data saturation | 22 | Was data saturation discussed? | n/a |
| Transcripts returned | 23 | Were transcripts returned to participants for comment and/or | n/a |

| **Topic** | **Item No.** | **Guide Questions/Description** | **Reported on**  **Page No.** |
| --- | --- | --- | --- |
|  |  | correction? | n/a |
| **Domain 3: analysis and**  **ﬁndings** | | | |
| *Data analysis* | | | |
| Number of data coders | 24 | How many data coders coded the data? | 14, 17, 18 |
| Description of the coding  tree | 25 | Did authors provide a description of the coding tree? |  |
|  |  |  | 14, 17, 18 |
|  |  |  |  |
| Derivation of themes | 26 | Were themes identiﬁed in advance or derived from the data? | 14, 17, 18 |
| Software | 27 | What software, if applicable, was used to manage the data? | n/a |
| Participant checking | 28 | Did participants provide feedback on the ﬁndings? | n/a |
| *Reporting* | | | |
| Quotations presented | 29 | Were participant quotations presented to illustrate the themes/ﬁndings?  Was each quotation identiﬁed? e.g. participant number |  |
|  |  |  | 14, 15, 16, 17, 18, 19, 20 |
|  |  |  |  |
| Data and ﬁndings consistent | 30 | Was there consistency between the data presented and the ﬁndings? | n/a |
| Clarity of major themes | 31 | Were major themes clearly presented in the ﬁndings? | 14, 17, 18 |
| Clarity of minor themes | 32 | Is there a description of diverse cases or discussion of minor themes? | 14, 15, 16, 17, 18, 19, 20 |

Developed from: Tong A, Sainsbury P, Craig J. Consolidated criteria for reporting qualitative research (COREQ): a 32-item checklist for interviews and focus groups. *International Journal for Quality in Health Care*. 2007. Volume 19, Number 6: pp. 349 – 357
